# Implementing CT tumor volume and CT pleural thickness into future staging systems for malignant pleural mesothelioma

**DOI:** 10.1186/s40644-021-00415-5

**Published:** 2021-08-03

**Authors:** Olivia Lauk, Miriam Patella, Thomas Neuer, Bianca Battilana, Thomas Frauenfelder, Thi Dan Linh Nguyen-Kim, Walter Weder, Claudio Caviezel, Sven Hillinger, Ilhan Inci, Isabelle Opitz

**Affiliations:** 1 Department of Thoracic Surgery, University Hospital Zurich, Raemistrasse 100, 8091 Zurich, Switzerland; 2 Institute of Diagnostic and Interventional Radiology, University Hospital Zurich, Raemistrasse 100, 8091 Zurich, Switzerland

**Keywords:** Malignant pleural mesothelioma, Computed tomography tumor volume, Pathological tumor weight, Macroscopic complete resection, Pleurectomy/decortication

## Abstract

**Objectives:**

Tumor thickness and tumor volume measured by computed tomography (CT) were suggested as valuable prognosticator for patients’ survival diagnosed with malignant pleural mesothelioma (MPM). The purpose was to assess the accuracy of CT scan based preoperatively measured tumor volume and thickness compared to actual tumor weight of resected MPM specimen and pathologically assessed tumor thickness, as well as an analysis of their impact on overall survival (OS).

**Methods:**

Between 09/2013–08/2018, 74 patients were treated with induction chemotherapy followed by (extended) pleurectomy/decortication ((E)PD). In 53 patients, correlations were made between CT-measured volume and -tumor thickness (cTV and cTT) and actual tumor weight (pTW) based on the available values. Further cTV and pT/IMIG stage were correlated using Pearson correlation. Overall survival (OS) was calculated with Kaplan Meier analysis and tested with log rank test. For correlation with OS Kaplan-Meier curves were made and log rank test was performed for all measurements dichotomized at the median.

**Results:**

Median pathological tumor volume (pTV) and pTW were 530 ml [130 ml – 1000 ml] and 485 mg [95 g – 982 g] respectively. Median (IQR) cTV was 77.2 ml (35.0–238.0), median cTT was 9.0 mm (6.2–13.7). Significant association was found between cTV and pTV (*R* = 0.47, *p* < 0.001) and between cTT and IMIG stage (*p* = 0,001) at univariate analysis. Multivariate regression analysis revealed, that only cTV correlates with pTV. Median follow-up time was 36.3 months with 30 patients dead at the time of the analysis. Median OS was 23.7 months. 1-year and 3-year survival were 90 and 26% respectively and only the cTV remained statistically associated with OS.

**Conclusion:**

Preoperatively assessed CT tumor volume and actual tumor volume showed a significant correlation. CT tumor volume may predict pathological tumor volume as a reflection of tumor burden, which supports the integration of CT tumor volume into future staging systems.

## Introduction

Malignant pleural mesothelioma (MPM) is an aggressive tumor with a median overall survival (OS) of 9 to 12 months [1]. It is strongly correlated with asbestos exposure with a long latency period [2, 3]. Despite the clear identification of this risk factor and the measures adopted to ban asbestos from construction industries, the MPM peak of incidence has not been reached yet, and it is expected in 2030 in developed countries [2]. Current guidelines [3–8] recommend a multimodality therapy approach in selected patients, consisting of induction chemotherapy followed by macroscopic complete resection (MCR) and/or adjuvant chemo- or radiotherapy. Surgical candidates must be carefully evaluated. Accurate clinical staging is at this point essential. The latter point is crucial and rather difficult to achieve, as the TNM staging system showed some flaws in clinical application. In fact, there is a poor correspondence between clinical and pathologic stage and the system itself lacks of prognostic significance, specifically for T1 and T2 disease. The use of strict criteria taking into account a precise segmentation of the CT scans by obtaining a surrogate for the tumor burden, has shown to have prognostic significance [9]. This is because the TNM is mostly based on surgical cases where the tumor invasion is assessed microscopically; in clinical practice, the distinction between parietal and visceral pleural involvement is not feasible with the current imaging modalities [10]. To overcome this problem, there has been a great interest in finding different clinical predictors that can serve as surrogate of the pathological measurement and help to stratify patients for enrollment in treatment protocols.

Quantitative assessment of the tumor burden has been evaluated as a potential predictor. Computed tomography (CT)-tumor volume has been proven to be a reliable prognosticator [11]. In contrast to most solid malignancies in which the mono-dimensional criterion is used, the volumetric analysis has shown to be more reliable in terms of inter- and intra-observer variability [12].

The aim of the present study was to assess the correlation of CT based tumor burden representative parameters with pathological measurement of the overall tumor burden.

## Patients and methods

We retrospectively analyzed CT scans as well as clinical and pathological records of patients treated for MPM at our institution from 09/2013 to 08/2018. The main objective of the study was to verify the correlation between CT descriptors and pathological findings in order to identify robust imaging derived clinical predictors of pathological measurements and which are available before surgery. Secondary end-point of the study was the correlation between CT descriptors and overall survival in our population.

All patients included underwent induction chemotherapy (platin-based chemotherapy/pemetrexed) followed by (extended) pleurectomy/decortication ((E)PD). Patients with incomplete data on CT descriptors, pathological descriptors and survival were excluded from the analysis. Local ethics committee approval was given for analysis of the mesothelioma database (StV 29–2009, EK-ZH 2012–0094). All patients signed written informed consent. All patient data was anonymized.

### CT-based tumor thickness and volume

All CT examinations were performed using different types of multidetector CT scanner (Somatom Flash, Somatom Force and Somatom Definition AS, Siemens, Erlangen Germany) before and after induction therapy. A standard chest CT protocol was applied with contrast agent. Section thickness was 2.5 mm with an increment of 2 mm. For the purpose of the study we only used the CT scan after chemotherapy/before surgery, for analysis median time between CT scan and surgery was 4 weeks.

CT descriptors were tumor thickness (cTT) and tumor volume (cTV). Tumor thickness was defined as the sum of the two thickest spots on axial section plane (perpendicular to the chest wall or mediastinum) each measured at three representative different levels (optimally at lower, middle, and upper level if feasible) given in mm, with at least measurements being performed at 2 different levels for each patient. cTV was derived semi-automatically on axial planes of the CT scan. The quantification of tumor volume was previously described by Frauenfelder et al. [12]. On every fifth to tenth CT section, respectively, the tumor contour was drawn semi-automatically by applying a live-wire algorithm using dedicated post-processing software (Myrian®; Intrasense, Paris, France). The contours in between were obtained by linear interpolation. The results were reviewed and were adjusted if necessary by two experienced chest radiologists (Prof. Frauenfelder and Dr. Nguyen-Kim). The software calculated the volume of the tumor by summarizing all voxels and multiplied by the voxel size.

### Pathology-based volume

Pathological samples were weighted directly in the operating room as well as volume was taken, before it was sent to the Institute of Pathology. Tumor volume was measured by assessing the specimen into a bowl filled with saline, the displaced fluid indicated the volume. Information on tumor weight (TW) are given in gram (g) and the total tumor volume (pTV) in milliliter (ml).

### Pathology-based staging

Pathological staging was assigned according to the 8th TNM classification and IMIG staging system [5] and for a better overview IMIG stage I and II were combined and referred to as “low stage” and stage III and IV as “high stage”. Overall survival (OS) was calculated from the start of chemotherapy to the date of death for any cause, with living patients censored at the date of last follow-up.

### Statistical analysis

Due to the very high correlation between pTV and TW (Pearson *r* = 0.94, *p* < 0.001), only pTV remained in the analysis to assess the predictability of the pathological variables given cTV and cTT, respectively.

Due to the non-normality of cTV and cTT the Kendall rank correlation coefficient was used to assess the association with pTV. The IMIG stage with levels I to IV was combined into a two-level factor. The stages I and II were combined into a “low” IMIG stage group, while stages III and IV were combined into a “high” IMIG stage group. This seemed sensible due to the low number of patients in some strata as well as for comparability with previous studies. Therefore a Wilcoxon-Mann-Whitney test was performed to assess its correlation with cTV and cTT.

Patient OS was assessed by Kaplan-Meier method. To assess the importance of pathological and clinical predictors separately two multiple Cox’s proportional hazard models were fitted to the data, firstly including pathological covariates pTV and IMIG stage, secondly including cTV and cTT. Two separate models were fitted in order to avoid highly correlated independent covariates in the same model and to observe which set of covariates explained survival times better.

Statistical significance was set at a *p*-value of *p* < 0.05. Data processing and analysis were performed with the statistical R-software version 3.6.2. The dataset supporting the conclusions of this article is included within the article. Additional data can be supplied on request.

## Results

Eighty-four patients underwent induction chemotherapy (platin-based chemotherapy/pemetrexed) followed by macroscopic complete resection (MCR) during the study period. Seventy-four patients underwent induction chemotherapy followed by (E)PD. In 53 patients all parameters (cTT, cTV, pTV, pTW, IMIG stage) were available for analyses. To be mentioned, that resected diaphragm and pericardium was included, in case of EPD in 49 patients. Patients’ characteristics are shown in Table 1. Median time between restaging CT or PET/CT and surgery was 24 days [1–90 days].

**Table 1:** Patient characteristics

Covariate	Overall (***n*** = 53)
**Male gender (%)**	48 (90.6)
**Asbestos (%)**	
no	17 (32.1)
possible	10 (18.9)
yes	26 (49.1)
**Smoking (%)**	(NA’s: 10 (18.9%))
current	6 (11.3)
former	21 (39.6)
never	25 (47.2)
unknown	1 (1.9)
**Packyear (median [Range])**	30 [0–100]
**Right laterality (%)**	36 (67.9)
**Type of surgery (%)**	
EPD	49 (92.5)
P/D	3 (5.7)
Extended parietal pleurectomy	1 (1.9)
**Response**	
Stable disease	32 (60.4)
Partial regression	14 (26.4)
Progressive disease	4 (7.5)
Unknown	3 (5.7)
**IMIG stage (%)**	
I (low)	5 (9.4)
II (low)	6 (11.3)
III (high)	36 (67.9)
IV (high)	6 (11.3)
**pT stage 8th edition (%)**	
1	5 (9.4)
2	7 (13.2)
3	35 (66.0)
4	6 (11.3)
**Pathological subtypes (%)**	
Epithelioid	38 (71.7)
Biphasic	14 (26.4)
Sarcomatoid	1 (1.9)
**Patient status (%)**	
alive	21 (39.6)
dead	30 (56.6)
lost follow up	2 (3.8)

*NS* Not significant

Response rate after induction chemotherapy showed in the majority of the cases a stable disease (*n* = 32) and partial response (*n* = 14). The pathological stage distribution, as seen in Table 1, showed 6 patients with postoperative stage IV which mainly was due to an intraoperative proven infiltration of the chest wall. As this was the case in a maximum of 1–2 spots, the decision was made for completion of the surgery and the resection margins at the chest wall were marked with clips intraoperatively. Overall, our macroscopic complete resection achievement was completed for 51 patients and 2 patients of missing data.

Median pTV and TW were 530 ml [130 ml – 1000 ml] and 485 mg [95 g – 982 g] respectively. Median (IQR) cTV was 77.2 ml (35.0–238.0), median cTT was 9.0 mm (6.2–13.7) (Table 2).

**Table 2:** CT-derived and actual measurements

Covariate	Overall
n	53 median [range]
Tumor volume (ml)	530 [130–1000]
Tumor weight (g)	485 [95–982]
CT tumor thickness (mm)	9 [3.1–39.8]
CT tumor volume (ml)	77.2 [1.5–787]
pre upper	6.85 [2–50.7]
pre middle	5.35 [1.5–33.8]
pre lower	7.7 [2.5–58.7]
pre max	10.8 [2.8–58.7]
pre sum	21.2 [8.6–90.6]
post upper	5.85 [1.7–37.3]
post middle	5.3 [1.8–29.4]
post lower	8.4 [2.2–39.8]
post sum	18.75 [8.3–72.2]
Tumor thickness thickest area	1.2 [0.2–7]

**Fig. 1:**
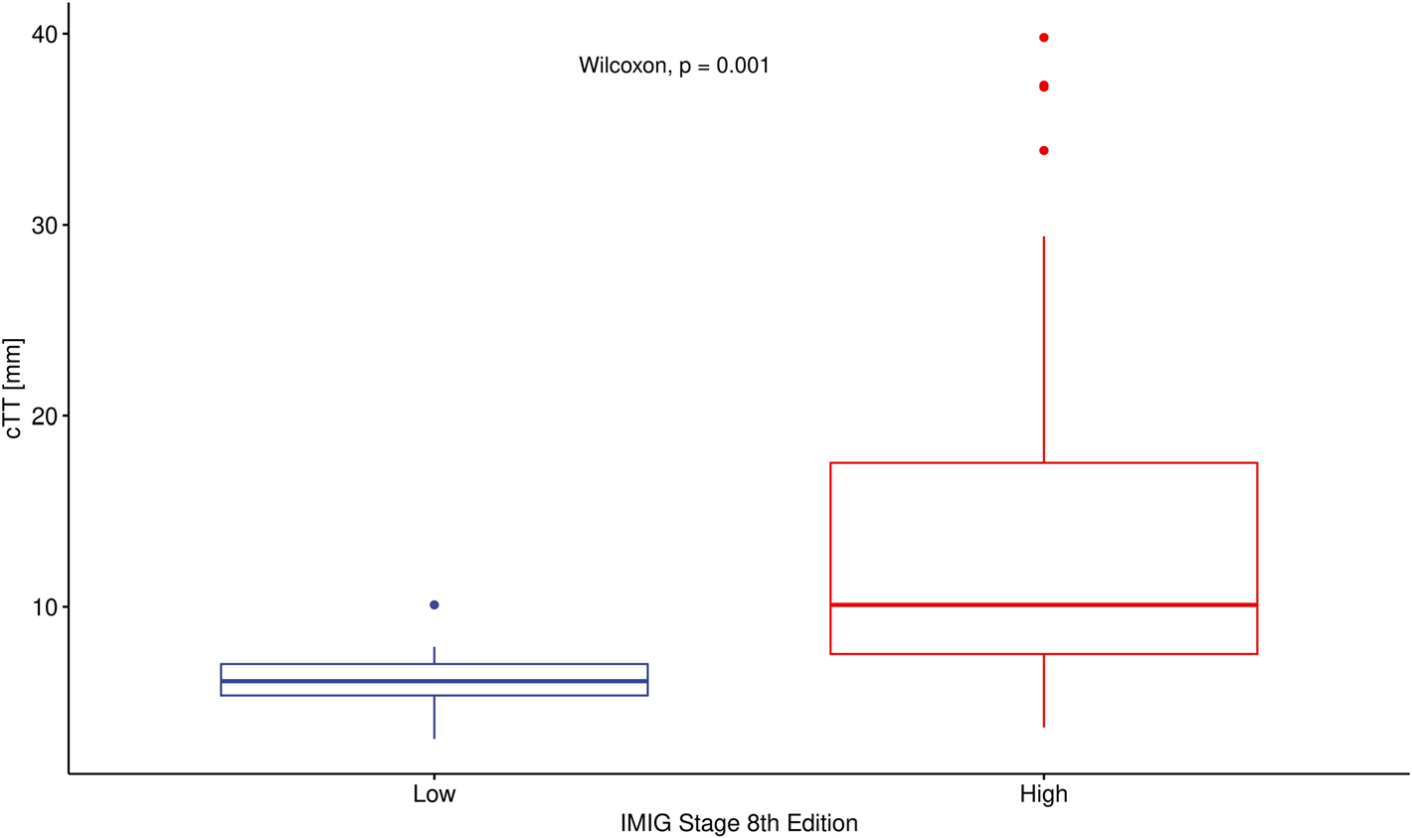
The distribution free Wilcoxon-Mann-Whitney test was used. IMIG stage low includes stage I and II, high includes stage III and IV. cTT (computed tomography derived tumor thickness)

**Fig. 2:**
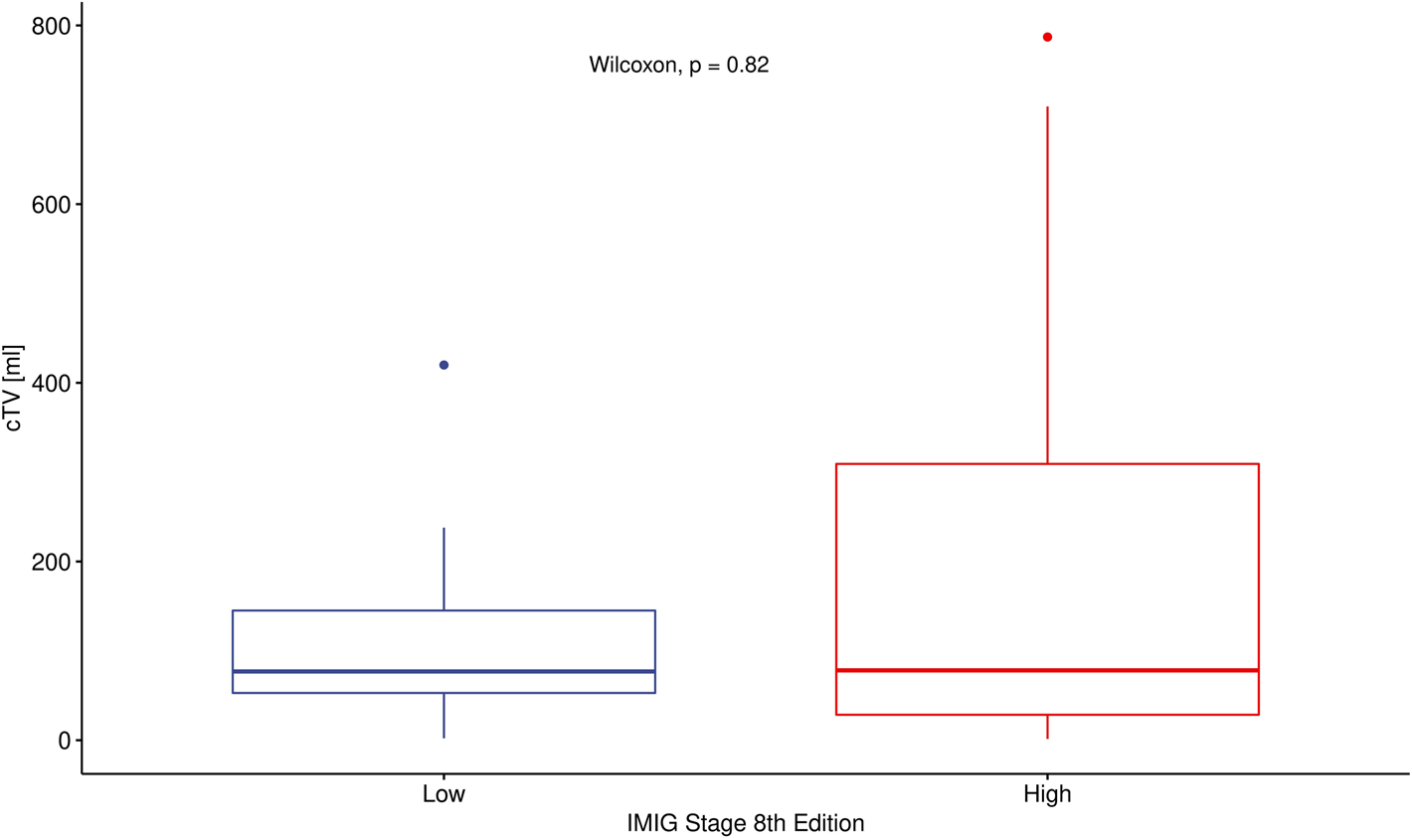
The distribution free Wilcoxon-Mann-Whitney test was used. IMIG stage low includes stage I and II, high includes stage III and IV. cTV (computed tomography derived tumor volume)

**Fig. 3:**
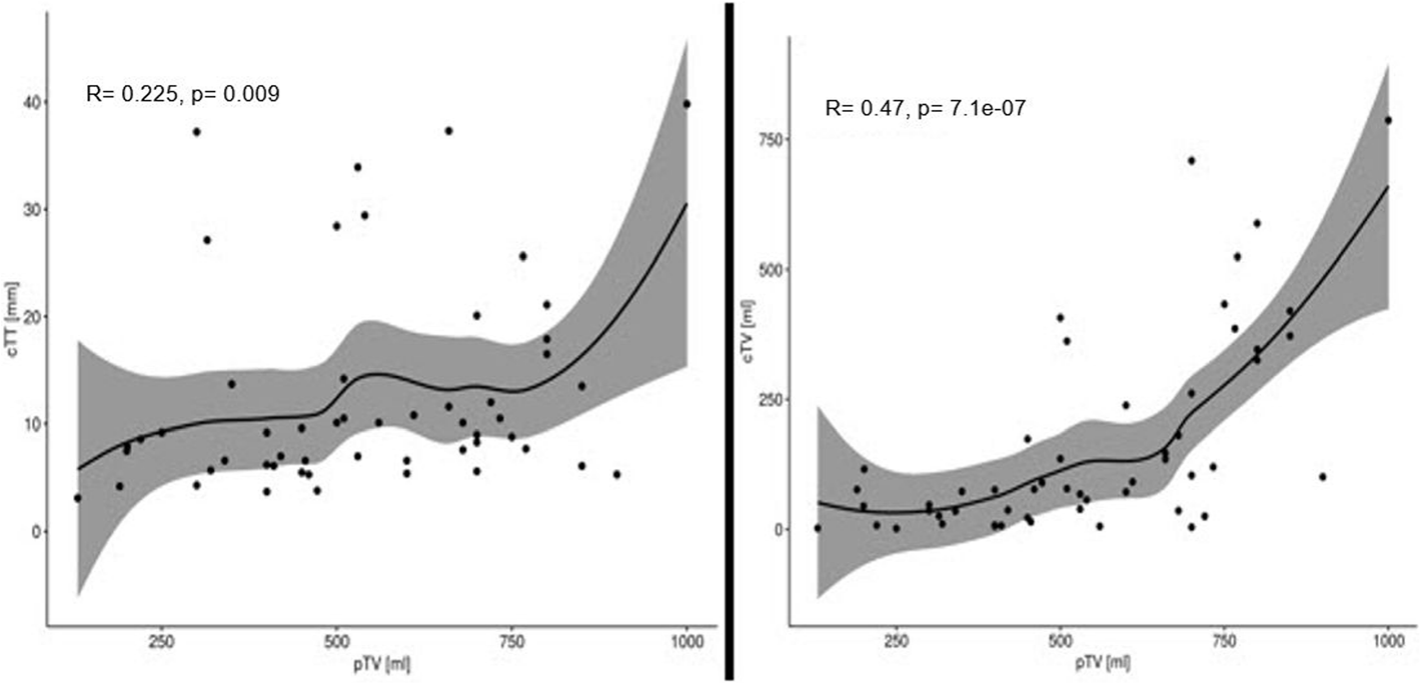
A non-parametric “loss” fit is shown as a linear relationship is not presumed. cTT = computed tomography derived tumor thickness, cTV = computed tomography derived tumor volume, pTV = pathological tumor volume

Significant association was found between cTV and pTV (*p* < 0.001) and between cTT and IMIG stage (*p* = 0,001) at univariate analysis. Further multivariate regression analysis including cTV and cTT confirmed, that only cTV remained correlated with pTV. Higher CT volume seems to be associated with higher pathological volume. Additionally, patients with higher IMIG stage have a higher tumor thickness. CT tumor thickness, does not result in additional information if CT tumor volume is given (*p* = 0,21) to predict pathological tumor volume as a reflection of tumor burden (Figs. 1, 2 and 3).

**Fig. 4:**
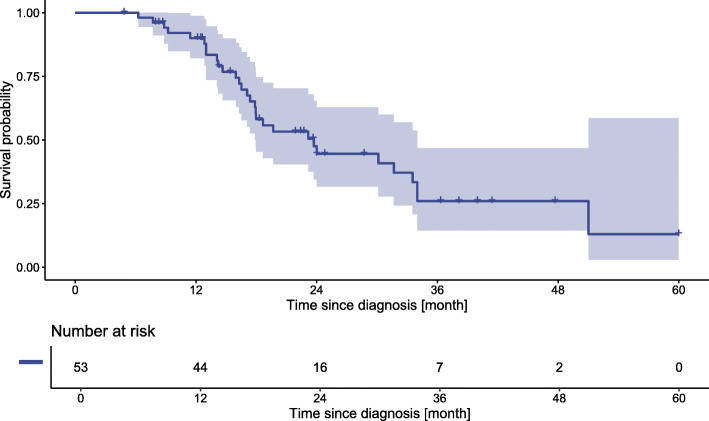
Median OS 23.7 months (CI 95%: LCL-UCL: 17.9–34)

Median follow up time was 36.3 months [CI 95% LCL: 23.6 months and UCL: 47.7 months] with 30 patients dead at the time of the analysis. Median OS was 23.7 months [CI 95% LCL: 17.9 months and UCL: 34 months] (Fig. 4), 1-year and 3-year survival were 90 and 26%, respectively.

When fitting two separate Cox Proportional-Hazards models for clinical and pathological covariates to the data, out of the four fitted parameters only the coefficient for cTV remained statistically significant (HR: 1.002, *p* = 0,02). The overall likelihood ratio test testing for improvements over the Null model without any covariates remained only significant for the model including clinical covariates (*p* = 0,003). This suggests a better fit for the clinical compared to the pathological covariates.

## Discussion

The present retrospective analysis of a surgical cohort being treated in a multimodality treatment concept showed a high correlation between preoperative assessed CT tumor volume and the actual tumor volume of the resected tumor specimen proposing a realistic reflection of CT data for preoperative decision-making. Furthermore, cTV was confirmed in alignment with previous studies to have a prognostic impact on OS. To our knowledge, pathological tumor weight and volume were assessed for the first time and being correlated with clinical measurements.

In 1998, Pass and colleagues [13] described a strong correlation between pre-operative CT tumor volume and prognosis being OS, progression-free survival and also with nodal spread. Moreover, they found an association between the cTV and the IMIG stage [13]. This was the first reported attempt of using the volumetric assessment for stratifying MPM patients. However, the manual tumor segmentation and the need for a time-consuming measurement, as well as the inter observer variability made the common use of cTV quite impractical. In 2010, Fan Liu and colleagues [14], benefitted from the use of semiautomatic computer method and imaging viewer system, for implementing a computer-aided tumor volume quantification. In their study, they grouped the TNM stages in “low stage” (I and II) and “high stage” (III and IV), finding a significant correlation between the cTV and the clinical TNM staging groups. However, this did not correlate when comparing the cTV with the pathological TNM stage. Nevertheless, they concluded that cTV variation after induction chemotherapy was a good predictor of the survival and computer-aided volume measurement could assist the radiologist in the precise tumor measurement. A group from the Harvard Medical School in Boston, embraced this principle and published two studies in 2012 [15] and 2018 [11]. The authors consistently focused on the prognostic performance of the cTV and they found a correlation between it and survival in MPM patients. In the second study [11], along with cTV, they evaluated in the same model, also a second descriptor of the MPM tumor burden: the maximal fissural thickness. After testing different models, they concluded that the combination of the cTV and the maximal fissural thickness performed better compared to the clinical AJCC staging even though there was no statistical difference in performance with the pathological AJCC staging [9, 12].

Overall, according to the literature and to our study results, pre-operative tumor burden may play a consistent role in the prognosis of patients with MPM. On the other hand, there is a consistent lack in correspondence between the clinical descriptors and the pathologic staging. This consideration supports the discussion around the evolving classification of the MPM in looking for incorporating different descriptors, to improve the clinical usefulness of the staging. Nowak at al [9], in their proposal for revisions of the T descriptors, proposed the measurement of pleural thickness as possible additional variable to incorporate into future staging. In our population, we found a correlation between cTT and IMIG stage, however, this variable failed to be retained in the multivariable analysis and showed to have no association with the OS. However, the combination of CT tumor volume and thickness showed a better result in potentially predicting survival time.

**Fig. 5:**
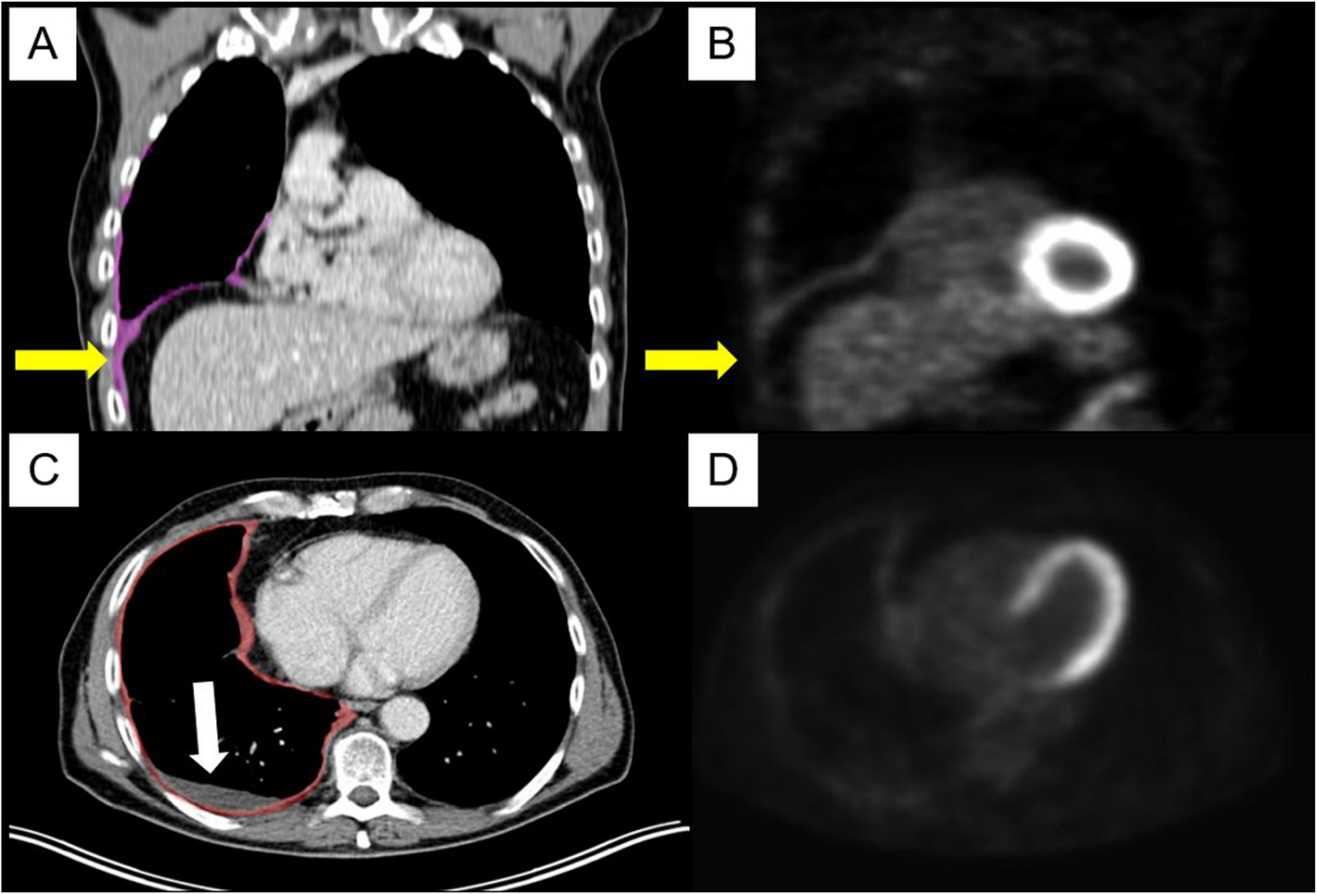
Segmentation of mesothelioma with simultanious correlation with Positron Emission Tomography (PET) images to distinguish from pleural effusion and/or atelectasis. **A**+**B**) Mesothelioma in lower part of pleural cavity with extension into the deep costophrenic recessus (yellow arrows). **C**+**D**) Pleural effusion (white arrow) without FDG activity in the PET images

It is important to underline that, unlike most solid tumors, which are suitable for a robust mono-dimensional measurement, the morphology of MPM, and almost constant presence of pleural effusion, may complicate the quantification (Fig. 5). Magnetic resonance imaging (MRI) for preoperative staging for patients with malignant pleural mesothelioma is more precise than CT derived clinical staging especially in context of the assessment for surgical resection because of its higher resolution for soft tissue and the evaluation of invasion of the thoracic endothoracic fascia, chest wall, diaphragm, and mediastinal fat. Because of these reasons the British Thoracic Society (BTS) recommends thoracic MRI in the decision making for a multimodality therapy approach including surgical resection [16]. Specificity and sensitivity of the MRI show an association with the tumor stage, where both are increasing with the higher tumor stage (87.5% for T2 and specificity of 91% and sensitivity of 100% for T3 stage) [17]. This may result in a general upstaging due to a better distinction of chest wall and mediastinal invasion and a better patient selection for a potential macroscopic complete resection [18]. The cTT may be better reflected on MRI. This may be one of the reasons for the lack of prognostic significance of cTT in our population due to the evaluation on a CT scan and additionally to the small sample size.

On the other side, CT imaging plays a historical role in malignant pleural mesothelioma staging as it is widely accessible and has a lower inter observer variability compared to MRI. Furthermore, CT scan is the most frequently used technique and radiologists have a broader experience in their evaluation. Although the extent of chest wall and/or mediastinal invasion is less accurate with CT derived imaging than MRI, intrathoracic lymphadenopathy and extrathoracic spread can be better detected, respiratory and cardiac motion artefacts are less frequently in CT than MRI [19]. Limitation of the MRI is the long acquisition time that are also the cause of the aforementioned artefacts [20].

When it comes to the assessment of CT derived tumor volume its role in preoperative evaluation is manifest [15]. As described previously, several groups proofed the correlation of cTV and T-status and their impact on OS and PFS [1]. They showed, that large volumes are associated with nodal spread as well as post resection residual tumor burden and that they may predict outcome [21, 22].

The missing impact of pTV and pTW on OS might be explained by the fact, that the tumor burden per se is not decisive as a prognosticator. It is also the type and localization of the tumor’s distribution as well as the lymph node involvement that may affect the patient’s survival.

Nevertheless, it has not been yet implemented in current staging systems due to inter observer variability and lack of reproducibility [16, 23, 24].

### Limitations

Our study has some limitations. Its retrospective nature carries an inherent bias. We have also to consider that our data are extracted from a purely surgical MPM population. Additionally, computed tomography generated T-stage suffers from the fact that in some cases not all measurements (lower, middle, upper) were available. Nevertheless, a minimum of measurements at two levels were required. This may result in a less representative value of tumor thickness measurement. Additionally, drawing any conclusion for the correlation of cTV and pTV is difficult, as the median volume of the specimen is much greater. This is due to the fact that resected diaphragm and pericardium, in case of EPD are part of it.

## Conclusions

In conclusion, our study supports the prognostic relevance of tumor burden in MPM patients, as it showed a high correlation between preoperative assessed CT tumor volume and the actual tumor volume of the resected tumor specimen. Computed derived tumor volume proposes a realistic reflection of CT data for preoperative decision making. The use of semi-automatic user-friendly software for accurate three-dimensional tumor evaluation is useful to implement CT descriptors in patients’ evaluation. This might improve the actual staging system and contribute to achieve a more accurate stratification of patients. Conformity between different medical centers might profit as well. Even if our results do not have the power for proposing a change in the staging system, they can serve as support for further studies.

## Data Availability

The dataset supporting the conclusions of this article is included within the article. Additional data can be supplied on request.

## Abbreviations

CT: Computed tomography
(E)PD: (extended) pleurectomy/decortication
cTV: CT-measured volume
cTT: CT -tumor thickness
pTV: Pathological tumor volume
pTW: Actual tumor weight
IQR: Median/Interquartile range
MPM: Malignant pleural mesothelioma
MRI: Magnetic resonance imaging
OS: Overall survival
